# Manual lesion segmentations for traumatic brain injury characterization

**DOI:** 10.3389/fnimg.2023.1068591

**Published:** 2023-03-16

**Authors:** Alexis Bennett, Rachael Garner, Michael D. Morris, Marianna La Rocca, Giuseppe Barisano, Ruskin Cua, Jordan Loon, Celina Alba, Patrick Carbone, Shawn Gao, Asenat Pantoja, Azrin Khan, Noor Nouaili, Paul Vespa, Arthur W. Toga, Dominique Duncan

**Affiliations:** ^1^USC Stevens Neuroimaging and Informatics Institute, Keck School of Medicine, University of Southern California, Los Angeles, CA, United States; ^2^David Geffen School of Medicine, University of California, Los Angeles, Los Angeles, CA, United States; ^3^Dipartimento Interateneo di Fisica “M. Merlin”, Università degli studi di Bari “A. Moro”, Bari, Italy; ^4^USC Department of Radiology, Keck School of Medicine, University of Southern California, Los Angeles, CA, United States

**Keywords:** segmentation, traumatic brain injury, epilepsy, magnetic resonance imaging, lesion mask, post traumatic seizures

## Abstract

Traumatic brain injury (TBI) often results in heterogenous lesions that can be visualized through various neuroimaging techniques, such as magnetic resonance imaging (MRI). However, injury burden varies greatly between patients and structural deformations often impact usability of available analytic algorithms. Therefore, it is difficult to segment lesions automatically and accurately in TBI cohorts. Mislabeled lesions will ultimately lead to inaccurate findings regarding imaging biomarkers. Therefore, manual segmentation is currently considered the gold standard as this produces more accurate masks than existing automated algorithms. These masks can provide important lesion phenotype data including location, volume, and intensity, among others. There has been a recent push to investigate the correlation between these characteristics and the onset of post traumatic epilepsy (PTE), a disabling consequence of TBI. One motivation of the Epilepsy Bioinformatics Study for Antiepileptogenic Therapy (EpiBioS4Rx) is to identify reliable imaging biomarkers of PTE. Here, we report the protocol and importance of our manual segmentation process in patients with moderate-severe TBI enrolled in EpiBioS4Rx. Through these methods, we have generated a dataset of 127 validated lesion segmentation masks for TBI patients. These ground-truths can be used for robust PTE biomarker analyses, including optimization of multimodal MRI analysis *via* inclusion of lesioned tissue labels. Moreover, our protocol allows for analysis of the refinement process. Though tedious, the methods reported in this work are necessary to create reliable data for effective training of future machine-learning based lesion segmentation methods in TBI patients and subsequent PTE analyses.

## 1. Introduction

Post traumatic epilepsy (PTE), which is defined as recurring seizures more than 1-week post-injury, can occur in up to 50% of patients with traumatic brain injury (TBI; Lowenstein, 2009). Despite the high incidence of PTE, the precise mechanisms which induce seizures are unknown due to the heterogenous nature of the disorder (Agrawal et al., 2006; Mukherjee et al., 2020). The onset of PTE is often preceded by a latency period, which can last up to several years (Garner et al., 2019). Currently, there are no effective interventions to prevent seizure development during this latency period. Therefore, identification of biomarkers to help predict PTE prior to seizure development is of great importance for early identification of at-risk patients. The Epilepsy Bioinformatics Study for Antiepileptogenic Therapy (EpiBioS4Rx) is a multi-center, international project which aims to identify biomarkers of PTE and utilize these biomarkers to develop effective and large-scale clinical trials for seizure prevention in TBI patients (Vespa et al., 2019). The characterization of injury burden, or size, location, and number of contusions, will facilitate biomarker identification. For example, recent studies have identified lesion characteristics such as lesion core and edema volumes, number of lesions, and lesion location in TBI patients as promising biomarkers for the development of PTE (Tubi et al., 2019; La Rocca et al., 2021).

Medical image segmentation is a useful tool for image processing, disease diagnosis, and prognosis (Patil and Deore, 2013; Norouzi et al., 2014). Specifically, lesion segmentation helps visualize and quantify injury burden. Accurate results from segmentation methods are necessary for robust analyses, especially in identifying reliable imaging biomarkers. Moreover, interpretation of automated medical image segmentation results can lead to biased downstream analyses, especially for smaller ROIs such as lesions (Müller et al., 2022). Manual segmentation methods, though tedious, allow for domain experts to accurately identify lesioned regions. In EpiBioS4Rx, we have designed a manual lesion segmentation process and performed it on structural MRI for 127 TBI patients to better characterize injury burden in a large sample size. Segmentations were performed on 3D T2 fluid attenuated inversion recovery (T2-FLAIR) images because the increased sensitivity to signal intensity often results with lesions presenting as hyperintense regions. A previous study noted the significance of hemorrhagic injury in the temporal lobe in seizure development after TBI. Other lesion types such as subdural hematoma, however, were not found to be associated with PTE (Tubi et al., 2019). Therefore, we segmented hemorrhagic, parenchymal contusions in this work. These gold standard ground-truths are important for training of automated segmentation methods and downstream PTE analyses.

Several automated segmentation methods for biomedical applications have been developed; however, TBI-induced deformations produce unexpected inconsistencies that can reduce accuracy in landmark-based segmentation algorithms (Irimia et al., 2012; Selvaganesan et al., 2019). Section 1.1, including advantages, limitations, and suggestions for future direction.

### 1.1. Review of automated segmentation methods

There has been a recent push in the development of methods to automatically segment lesions in TBI patients. These methods have the potential to alleviate the burden of manual segmentations, which are tedious, time consuming, and require neuroimaging expertise. Unsupervised segmentation will be very useful in the analysis of large datasets, which is necessary to generalize findings in a population. However, more reliable methods must be developed and validated to maximize clinical relevancy.

Toolboxes such as the Lesion Segmentation Tool (LST) have been used for an array of biomedical applications for its ability to segment hyperintense lesions in FLAIR images of multiple sclerosis patients (Schmidt and Wink, 2017). However, this is not optimized for the complexities present in TBI patients such as lesion size, location, and intensity differences. Therefore, popular toolboxes fail when tasked with segmenting TBI-induced lesions. FreeSurfer is another commonly used tool for automated medical image segmentation (Fischl et al., 2002). Similarly, this tool becomes less reliable in lesioned brains as it is based on a T1-weighted structural image (Selvaganesan et al., 2019). While it is important to note T2 or FLAIR images can be added in FreeSurfer for improved pial surface reconstruction, both focal and global lesion-induced error can still greatly impact brain morphometry measurements (King et al., 2020). Therefore, some studies have proposed post-processing methods to account for lesion induced errors. For example, Diamond et al. (2020) described a lesion correction method used to correct cortical volume measurements in patients with traumatic brain injury. Other methods exclude lesioned ROIs from FreeSurfer segmentation methods (Drijkoningen et al., 2017). However, such methods still have limitations, including the need for manual editing and the assumption of only focal lesion-induced errors.

Due to the limitations of using these popular segmentation algorithms, several recent studies have proposed novel machine learning methods specific to TBI populations. Many of these works have focused on lesion segmentation of computed tomography (CT) scans of TBI patients. For instance, a 2D deep learning architecture was utilized for CT images from 45 TBI patients which resulted in a dice similarity coefficient (DSC) value of 64% when outputted masks were compared to manually segmented ground-truths (Remedios et al., 2019). DSC has been used throughout literature as a method to statistically validate medical image segmentations based on spatial overlap (Zou et al., 2004). DSC values can range from 0 (no spatial overlap) to 1 (complete spatial overlap). While DSC is a commonly used metric for binary segmentation validation, there are limitations. For example, it is not robust in smaller regions (Zou et al., 2004), which may affect analyses of small brain lesions. Until recently, lesion type has also rarely been accounted for in automated frameworks (Phaphuangwittayakul et al., 2022); however, subtypes of lesion may provide invaluable information in the characterization of PTE. Inkeaw et al. (2022) used a CT scan integrated with bone window as an input to a deep learning model for segmenting three hemorrhage subtypes in TBI patients. However, sensitivities for each subtype ranged only from 35 to 58% (Inkeaw et al., 2022). In a different study, Phaphuangwittayakul et al. (2022) proposed a fine-tuned EfficientNet-B2 model for CT scans that outperformed baseline models in detecting lesion subtypes.

While several proposed methods have been applied to CT, MRI offers better tissue resolution and is suggested to be more accurate for PTE characterization (Garner et al., 2019). This is an important consideration when developing machine learning models, as it may improve accuracy. Kamnitsas et al. (2017) proposed a 3D convolutional neural network (CNN) for segmentation of TBI lesions, brain tumors, and ischemic stroke lesions from MRI. The model outperforms state-of-the-art architectures on brain tumors and ischemic stroke lesions, with DSC values of 89.8 and 66%, respectively. However, the results were lowest for TBI lesions, with a DSC value of 63% when compared to manually segmented ground-truths (Kamnitsas et al., 2017). Similarly, a random forest framework for contusion segmentation of MR images reached a mean DSC value of 60% (Rao et al., 2014). This captures the particularly difficult nature of developing automated methods in TBI patients.

Further, current methods in literature do not focus on the separation of lesion core to edema volumes, and instead segment total lesion volumes. While total volume is of great importance, subtle differences between lesion core and edema volumes may provide a better characterization of PTE as we previously reported a statistically significant difference in the ratio of lesion core-to-edema volume in seizure and non-seizure groups (Bennett et al., 2022a,b). Therefore, future works investigating lesion characteristics as PTE biomarkers should consider automated methods with the ability to segment each region of interest separately. One recent study presented the first neural network to distinguish between blood, core, and edema during automated segmentation, but reached a maximum DSC value of 53.9% (Rosnati et al., 2022). Another study derives lesion volumes for multiclass hemorrhagic lesions and edema using a deep learning model on CT scans (Monteiro et al., 2020). After exclusion of small lesions (<1 ml), the median DSC value for intraparenchymal hemorrhage and perilesional edema were 65.2 and 44.8%, respectively (Monteiro et al., 2020). These studies highlight the need for further work in the development of accurate models with the capability of separating lesion core and edema volumes, as this multiclass segmentation may allow for better prognostication. Here, we generate a gold-standard ground-truth dataset of lesion segmentation masks with binarized labels for lesion core and edema, which can be used to improve automated multiclass segmentation methods in future works.

## 2. Materials

### 2.1. Participants

This study was approved by the University of California, Los Angeles Institutional Review Board and the local review boards at each EpiBioS4Rx Study Group institution. At the time of writing, there are 250 subjects currently enrolled in EpiBioS4Rx from 13 international sites. Patient demographics for the EpiBioS4Rx cohort and the subset of patients with available lesion segmentations are detailed in Table 1. All patients have been admitted within 72 h of sustaining a moderate-severe TBI. Additional enrollment criteria for the study are outlined in Table 2. Eleven subjects withdrew from the study and 42 died prior to the 2-year follow up. Of these 53 subjects, quality was deemed usable in 17 patients, and lesion segmentation masks are still available for these subjects for ground-truths in TBI patients; however, they are excluded from any seizure analyses.

**Table 1:** Demographics for entire EpiBioS4Rx cohort and subset of patients with completed lesion segmentations.

**EpiBioS4Rx cohort**		**Completed lesion segmentation cohort**	
**Variable**	**Value**	**Variable**	**Value**
Age	44.28, SD = 21.2	Age	43.27, SD = 20.18
Sex	192 male/58 female	Sex	98 male/29 female
Arrival GCS	7.89, SD = 3.91	Arrival GCS	8.03, SD = 3.98
MRI post-injury date	12.16, SD = 12.54	MRI post-injury date	11.81, SD = 7.74

**Table 2:** Enrollment criteria for EpiBioS4Rx.

**Inclusion criteria**	**Exclusion criteria**
Acute traumatic brain injury	Diffuse axonal injury without hemorrhagic contusions
Intracranial, cortical, and/or subcortical bleed on CT imaging	Known HIV/AIDS, Hepatitis B or C
Age 6–100	Pregnancy
Glasgow coma score 3–13	Pre-existing neurologic disease, CNS malignancy, epilepsy/seizure disorder, or dementia
Enrollment within 72 h of injury	Isolated anoxic brain injury
Ability to undergo continuous EEG monitoring 7 days post injury	Devastating cervical spine injury
Ability to undergo MRI within 18 days post injury	Brain death
Ability to remain in study for 2 years	Present or pending incarceration
	Positive COVID-19 test

**Figure 1:**
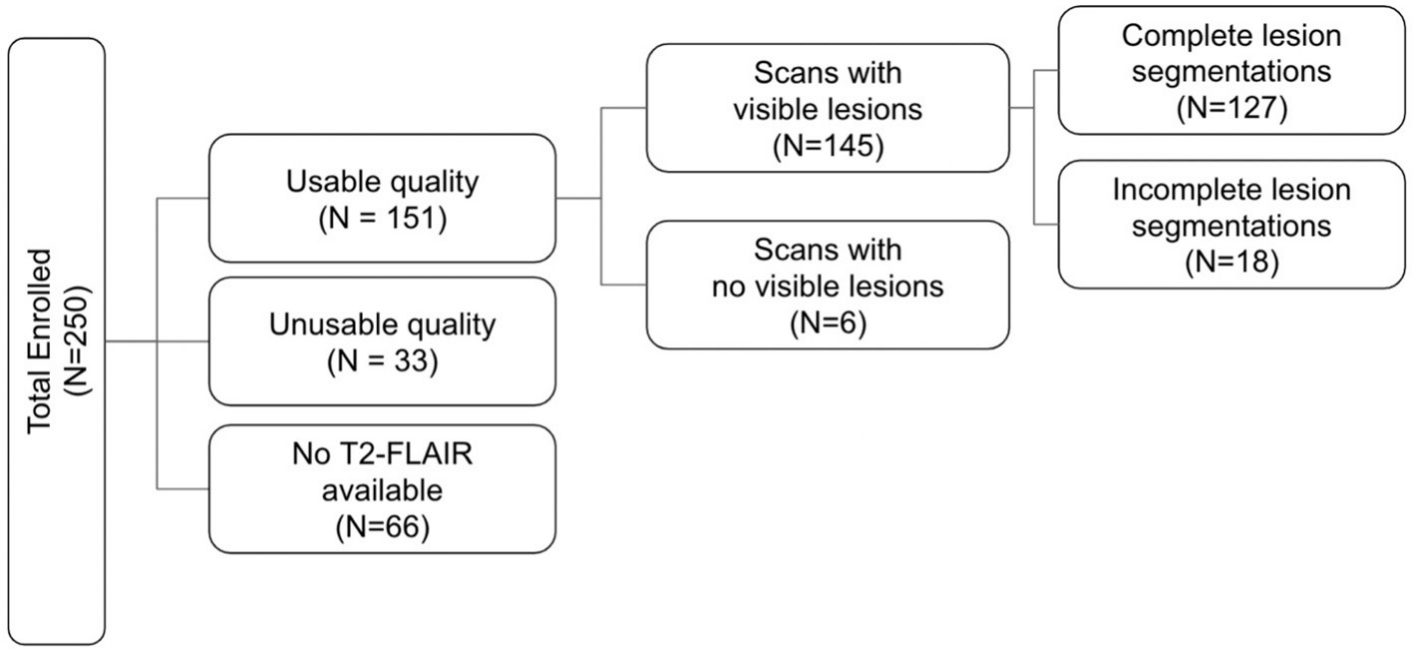
Flowchart outlining the amount of data from EpiBioS4Rx used at the time of writing.

In this work, we report methods validated on 133 patients enrolled in EpiBioS4Rx, 6 of which had no visible lesions. Therefore, 127 validated lesion segmentation masks are currently available (Figure 1). The remaining subjects were not included in this work due to poor quality data or if a T2-FLAIR sequence was not acquired or uploaded to the central repository at USC.

### 2.2. Data acquisition

Structural MRI volumes were acquired on 3-Tesla or 1.5 Tesla scanners according to the EpiBioS4Rx protocol outlined in Vespa et al. (2019). 3D T2-FLAIR volumes were acquired ~2 weeks post-injury (mean = 11.81, SD = 7.74). The following parameters were used: slice thickness = 1 mm, field of view = 256 mm, frequency = 256 Hz, flip angle = 90°/≥ 120°, T_R_ > 5,000 ms, T_E_ = 80–140 ms, T_I =_ 2,000–2,500 ms, gap = 0 gap, isotropic = 1 mm, NEX ≥ 1, up to 2x parallel imaging.

3D T1-weighted volumes were acquired using a magnetization prepared rapid acquisition gradient echo (MPRAGE) sequence. The following parameters were used: slice thickness = 1 mm, field of view = 256 mm, frequency = 256 Hz, flip angle = 8–15°, T_R =_ 1,500–2,500 ms, T_E =_ Min, T_I_ = 1,100–1,500 ms, gap = 0 gap, isotropic = 1 mm, NEX ≥ 1, up to 2x parallel imaging.

## 3. Methods

### 3.1. Manual lesion segmentation using ITK-SNAP

Anonymized MRI scans were examined both through visual inspection and the Laboratory of Neuro Imaging (LONI) quality control (QC) system to ensure high quality data were being used (Kim et al., 2019). After QC, T2-FLAIR volumes were uploaded into ITK-SNAP, a software used for medical image segmentation (Yushkevich et al., 2006). Image heterogeneity and noise can sometimes render it difficult to visualize contusions. In these cases, T1-MPRAGE sequences were uploaded as an additional reference image.

**Figure 2:**
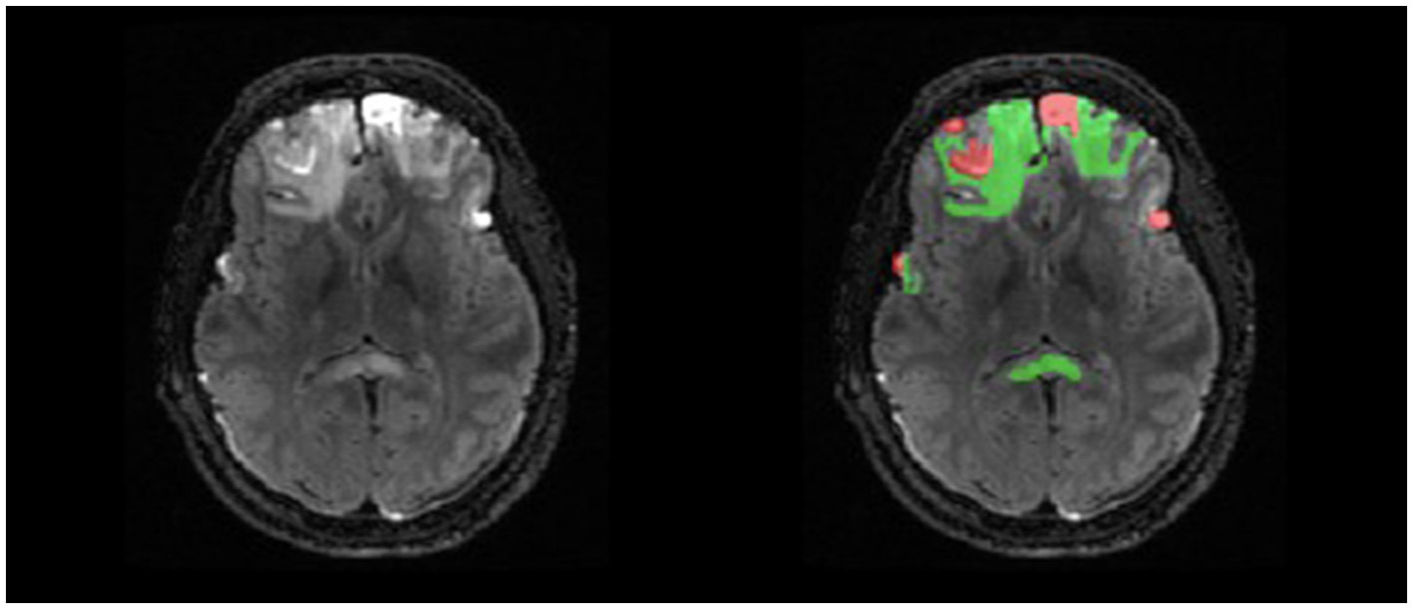
Example of double-reviewed completed parenchymal lesion segmentation on T2-FLAIR. Lesion core is depicted by the red label and surrounding edema is depicted by the green label.

Manual delineations were completed for parenchymal hemorrhagic contusions, with two independent labels for lesion core and surrounding edema (Figure 2). The labels were defined using the following guidelines: lesion core is a region with abnormal signal intensity and >1 ml of hemorrhagic volume and edema is the surrounding area of hyperintense signal (Chang et al., 2006; Iaccarino et al., 2014; La Rocca et al., 2021). Manual traces were completed on each lesioned slice of the T2-FLAIR in the axial plane to remain consistent across raters and produce a smoother 3D mask. Finally, we used label thresholding using FMRIB Software Library (FSL; Jenkinson et al., 2012) to generate binarized masks of lesion core, edema, and total lesion volume (lesion core + edema), allowing for future analyses of specific ROIs.

### 3.2. Training protocols

Segmentations were initially performed by five student researchers with minimal neuroimaging or segmentation experience. Our preliminary work which defines lesion and edema guidelines for this study (La Rocca et al., 2021) was sent to students as part of an initial onboarding process. A segmentation demonstration using ITK-SNAP exposed students to MRI, lesion visualization, and the software. Additionally, a comprehensive lesion segmentation guide was distributed for future reference (Supplementary material). To enforce guidelines on a practical level, biweekly check-ins with senior staff researchers with 3+ years of neuroimaging and segmentation experience were held for consistent feedback.

### 3.3. Dataset validation

**Figure 3:**
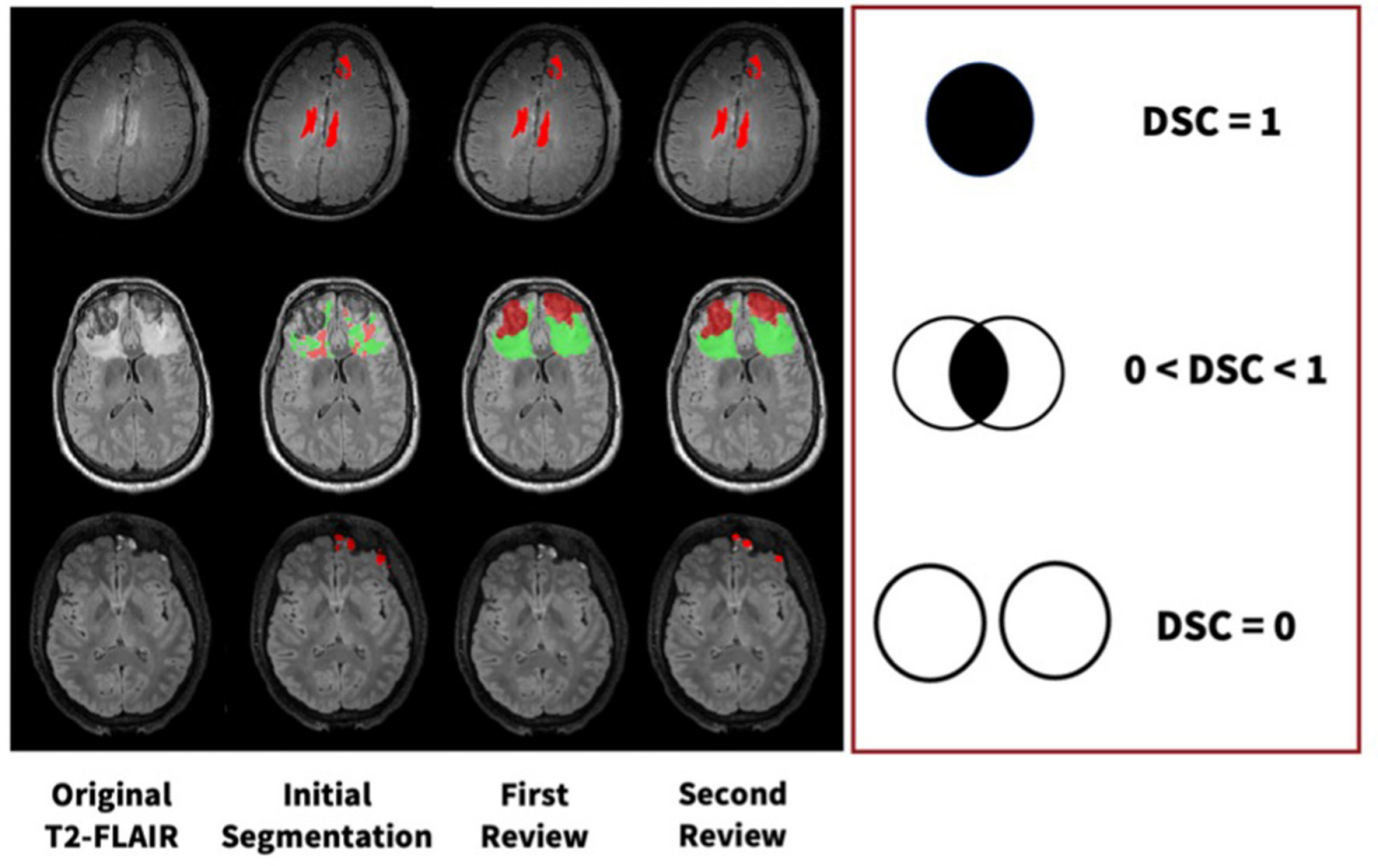
Examples of manual segmentation process for three subjects at each step of our manual review process with varying levels of DSC values.

All segmentation masks were first reviewed and edited as needed by either senior staff researchers with 3+ years of neuroimaging experience or medical doctors. To validate these data, a final review was completed by another physician with neuroradiology expertise. More specifically, the reviewer had domain expertise in neurocritical care. These double-reviewed segmentations are then used for ground-truths in all subsequent analyses and algorithm training. We calculated DSC values at each step of the review process, which can be visualized in Figure 3. Using DSC values for voxel overlap between the first and second reviews, we established that majority of change occurs during the first review.

## 4. Results

### 4.1. Dice similarity comparisons of refinement process

**Figure 4:**
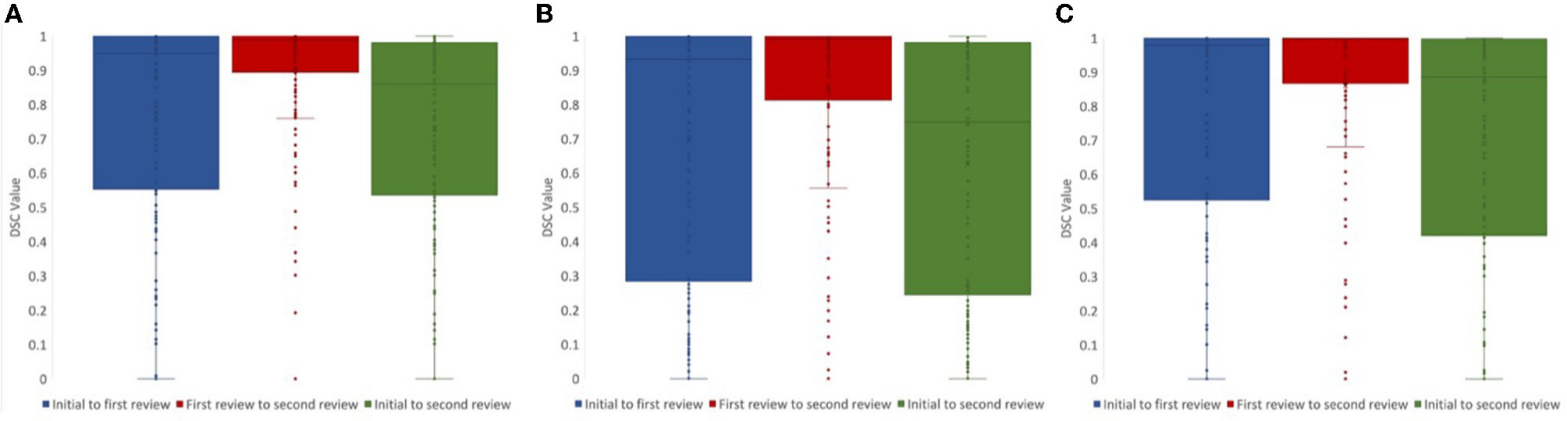
DSC values comparing the review steps in a manual lesion segmentation process for **(A)** total lesion, **(B)** core, and **(C)** edema. The difference between initial segmentation to first review (blue) and first review to second review (red) were significantly different for all ROIs (*p* < 0.05). The difference between initial segmentation to second review (green) and first review to second review (red) were also significantly different for all ROIs (*p* < 0.01).

**Figure 5:**
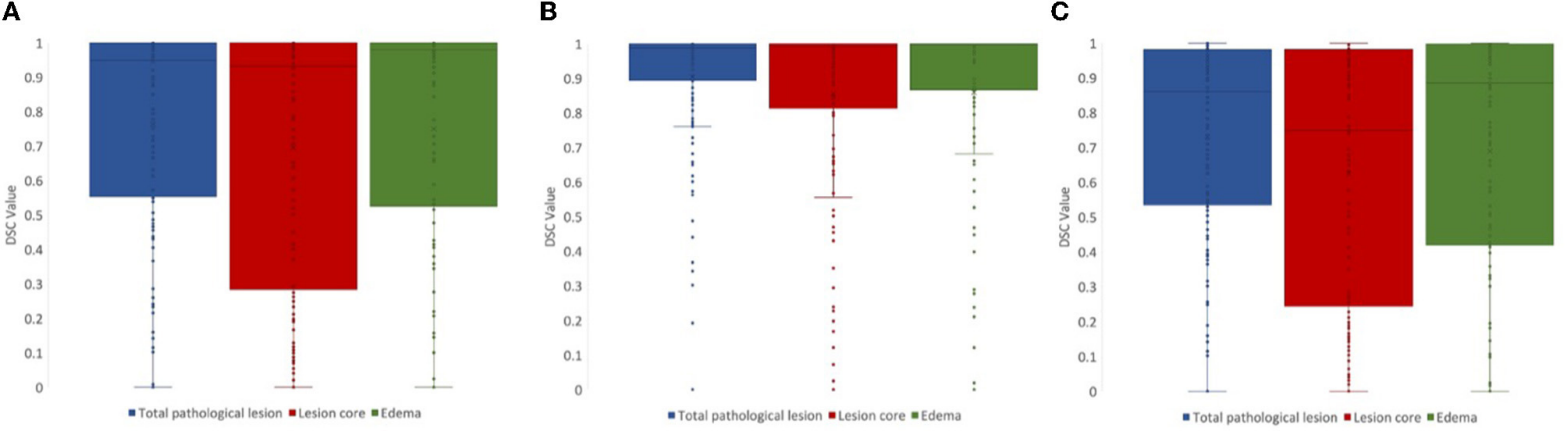
DSC values for **(A)** initial segmentation to the first review and **(B)** first review to the second review were not significantly different between any ROIs (*p* > 0.05). DSC values for **(C)** initial segmentation to the second review were significantly different between lesion core (red) and total lesion (blue; *p* < 0.05).

For lesion core volume, edema volume and total lesion volume, the average DSC value between the first and second review was 0.86, 0.86, and 0.91, respectively. DSC values at different steps in the review process were tested using a one-way ANOVA test, and Tukey's HSD test was used for *post-hoc* analysis. We compared DSC values for the following steps: (1) initial segmentation to first review, (2) first review to second review, and (3) initial segmentation to second review. For lesion core volume, edema volume and total lesion volume, the average DSC value between the initial segmentation and first review was 0.77, 0.74, and 0.74, respectively. For lesion core volume, edema volume and total lesion volume, the average DSC value between the first and second review was 0.86, 0.86, and 0.91, respectively. DSC values were found to be significantly lower for the initial segmentation to first review than the first review to the second review for all ROIs (*p* < 0.05; Figure 4). Further, DSC values for the initial segmentation to the second review were significantly different between lesion core and total lesion (*p* < 0.05; Figure 5). However, there was no significant difference between lesion core and total lesion for the other review steps. Moreover, there was no significant difference between lesion core and edema at any review step.

### 4.2. Expected outcome of manual segmentation protocol

**Figure 6:**
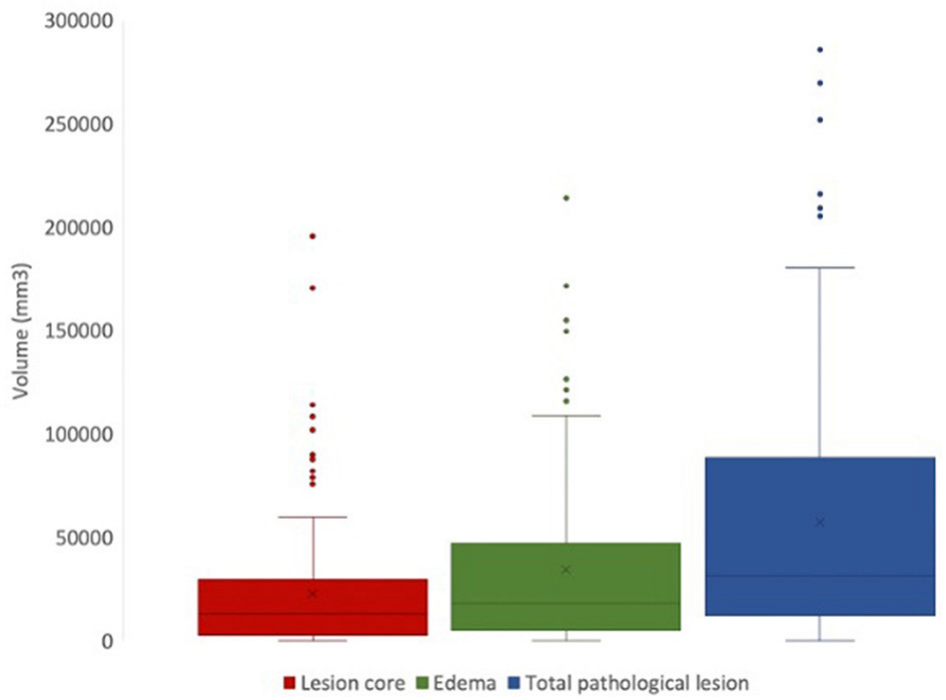
Total volumes of lesion core (red), edema (green), and total pathological lesion (blue) for 127 EpiBioS4Rx subjects used in this study.

Using FSL, we can extract lesion characteristics from manually generated masks that can be used for downstream analyses (Figure 6). From the available 127 lesion masks, the average lesion core volume was 23304.8 *mm*^3^(SD = 31703.52). The average edema volume was 35726.38 *mm*^3^ (SD = 41375.71). The average total pathological lesion volume across all subjects was 59031.18 *mm*^3^ (SD = 62726.89).

**Figure 7:**
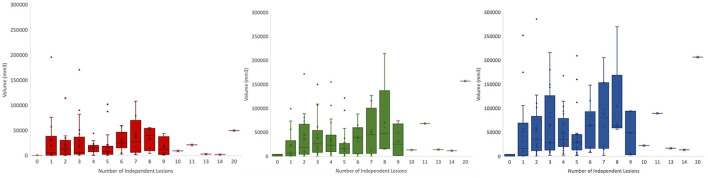
Total volumes for lesion core **(left)**, edema **(middle)**, and total pathological lesion **(right)** for each lesion number found in the cohort.

This dataset captures the heterogeneity associated with TBI-induced lesions. Figure 7 displays core, edema, and total pathological lesion volumes for each number of independent lesions found in this cohort. Through visual inspection, we found a range of 0–20 independent lesions (average = 4.32, SD = 2.92) in this cohort. However, there is large variability in volumes across patients. For instance, in patients with 1 lesion, total pathological lesion volumes range from 308.98 to 251777 *mm*^3^, with an average volume of 51298.16 *mm*^3^ (SD = 76047.66).

## 5. Discussion

### 5.1. Advantages of manual lesion segmentation

**Figure 8:**
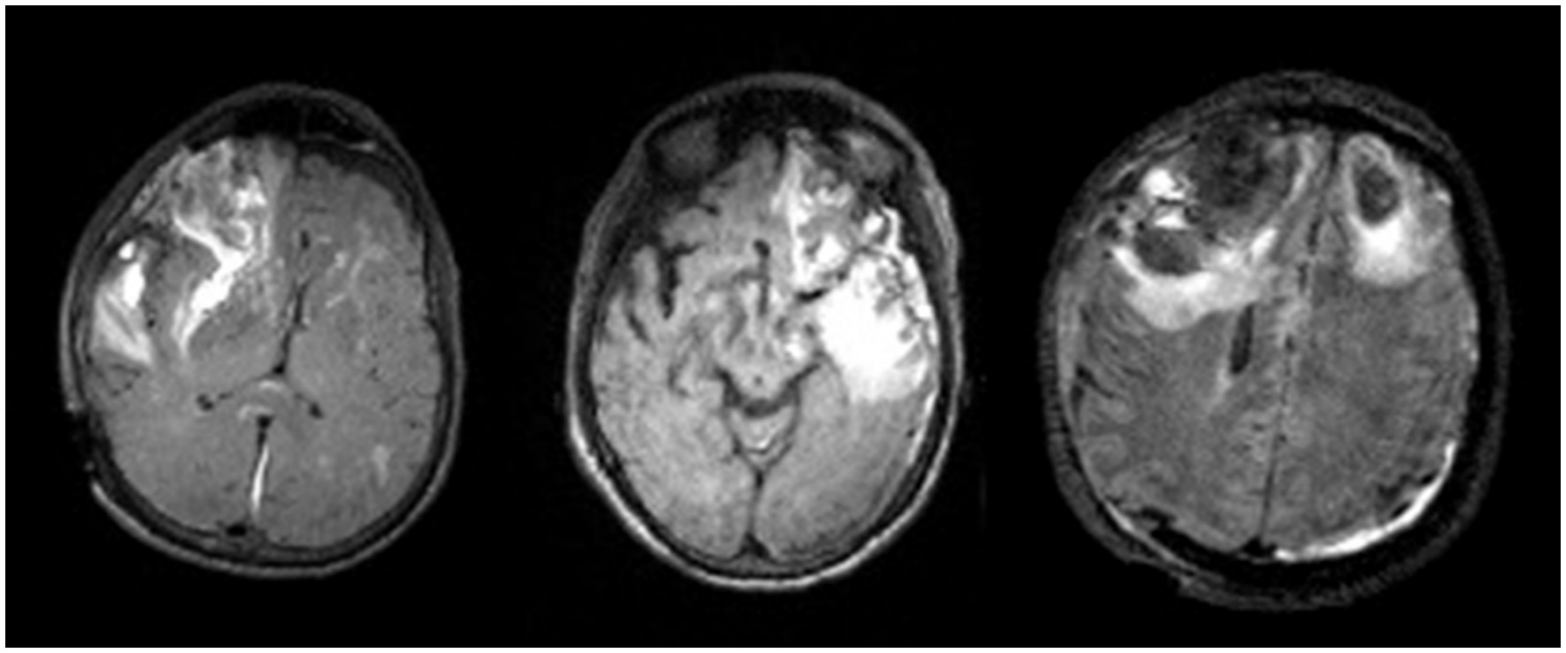
Visualization of lesion heterogeneity in axial slice of 3 TBI subjects.

Importantly, lesion segmentation ground-truths for a large population of patients with TBI have been generated through this work. Many models in literature are trained on limited sample sizes, which can be attributed to difficulties in acquiring ground-truths. However, the substantial sample size of ground-truth segmentations we have generated for TBI subjects will help to improve upon current methods. In this heterogeneous dataset, there are subtle differences that can reduce accuracy in automated methods. For instance, the lesion core can most often be visualized as a hyperintense region on a T2-FLAIR volume. However, Figure 8 shows that in some patients, the lesion core can appear as a hypointense region, or it can be difficult to distinguish the border between core and edema. Differences due to injury, noise, and acquisition techniques can play a large role in inter-subject variation. Moreover, deformations can cause preprocessing techniques to fail in TBI cohorts. Therefore, capturing more of the potential heterogeneity of injuries in our broad, multi-site dataset is ideal for the development of a pipeline that does not require robust preprocessing. Through manual segmentation, we have curated labels to account for injury complexities and improve the learning of lesion features in automated methods.

### 5.2. Limitations, possible pitfalls, and associated solutions

**Figure 9:**
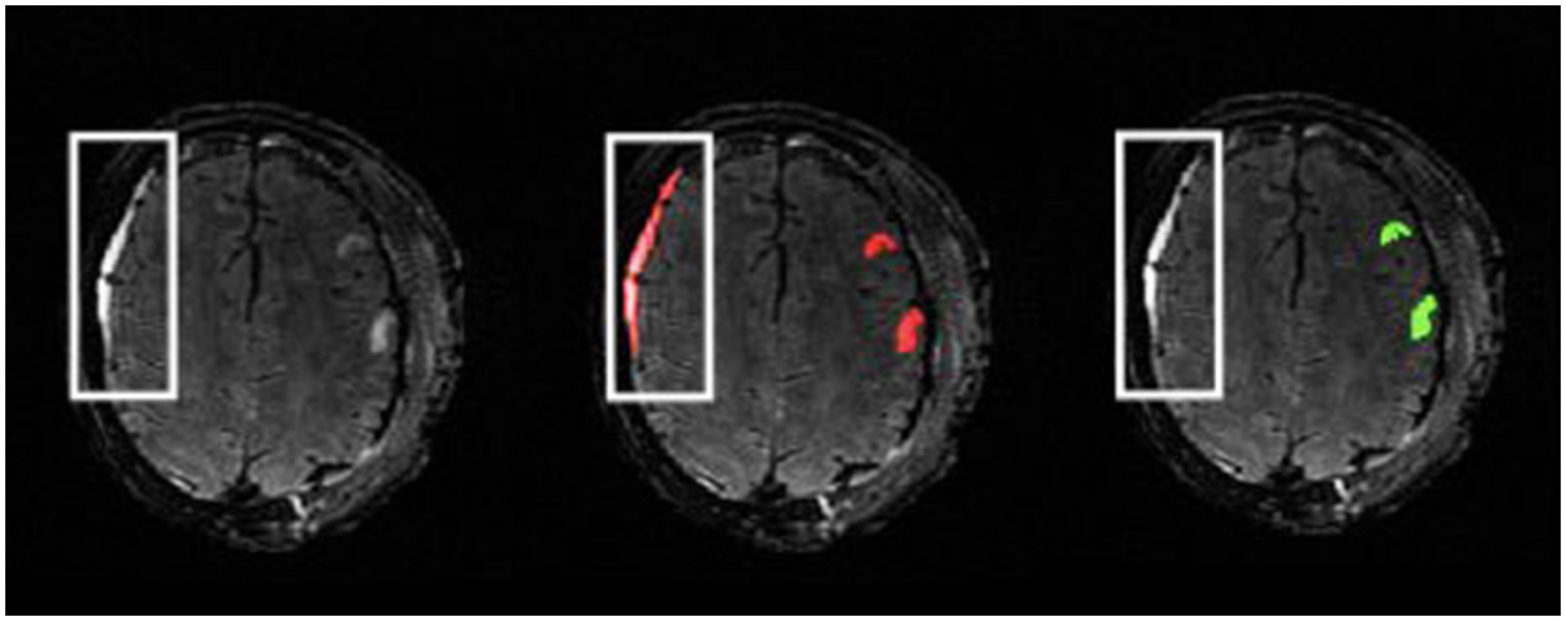
Example of subject with subdural hematoma presenting as a hyperintense region in the outlined area on a T2-FLAIR **(left)**. The initial segmentation includes this as lesion core **(middle)**. The reviewed ground-truth **(right)** excludes this region from the parenchymal lesion segmentation.

The described manual lesion segmentation process is very time intensive, which is a major limitation. The potential lifecycle of the review process for one complete segmentation mask can range from hours to weeks, especially for patients with extensive injuries. The length of this process is dependent on student experience, lesion volumes, and availability of domain experts. As a result, it can be difficult to recruit qualified personnel to annotate and review MRI scans. Even still, the manual work performed is necessary to lay groundwork for robust MRI analysis with accurate tissue segmentation as well as future PTE studies relating to lesion characteristics. We trained and supervised high school and undergraduate students to perform lesion segmentations. This alleviates the time commitments of field experts, which makes the generation of such a dataset more accessible. However, for those with minimal experience in neuroimaging and medical image segmentation, there is of course a steep learning curve for interpreting MRI scans, especially in TBI cohorts due to lesion heterogeneity. Distinguishing lesion core from edema in some images can be difficult. Moreover, this work describes a protocol for the segmentation of parenchymal contusions. Subdural hematomas can also appear as hyperintense regions on T2-FLAIR images (Oshida et al., 2019) and were commonly segmented as parenchymal lesion core by students at first (Figure 9). Learning a new software for image segmentation can also hinder performance. However, to account for this we have an initial onboarding session to provide an overview of ITK-SNAP, MRI, and lesion segmentation. We also provide a comprehensive tutorial document with examples of routine and complex cases to each student afterwards for future reference (Supplementary material).

Additionally, this study focuses on the segmentation of only intraparenchymal hemorrhagic lesions and the associated perilesional edema. Other lesion types, such as traumatic subarachnoid hemorrhage, were not included in this study due to the time-consuming and difficult nature of manually segmenting these lesion types, especially in cases with extensive subarachnoid hemorrhage. Moreover, EpiBioS4Rx aims to identify reliable imaging biomarkers from multiple modalities (Vespa et al., 2019). The segmentation masks generated in this study allow for more accurate analyses of fMRI and diffusion-derived maps as these parametric maps are typically from intraparenchymal regions. However, the future addition of extraparenchymal lesions may help identify additional biomarkers of PTE, especially as traumatic subarachnoid hemorrhage has been identified as a potential contributor to seizure development after TBI (Fordington and Manford, 2020; Laing et al., 2022).

### 5.3. Analytical methods derived from data

From this rich dataset, we can investigate these lesion characteristics on individual and group levels for in-depth analyses for PTE biomarker identification and validation. For instance, a preliminary report of manually segmented lesion masks from 32 subjects enrolled in EpiBioS4Rx revealed a relationship between lesion volume and the onset of seizures after TBI (La Rocca et al., 2021). Moreover, there was a correlation between positive seizure outcomes and lesions in the limbic parahippocampal gyrus and sub-lobar regions (La Rocca et al., 2021), highlighting the importance of manual lesion segmentation in PTE biomarker identification. Subtle lesion differences, such as number of independent lesions and ratio of lesion core to edema, have also been identified as potential indicators of seizure onset (Bennett et al., 2022a,b). These statistical analyses offer great insight into lesion characteristics of interest.

Most importantly, the methods outlined provide a large dataset of ground-truth manual segmentations, which is important to increase statistical power in PTE analyses and increase sample size for training machine learning models for segmentation tasks. Training and validating machine learning models with the data is an overarching goal. Another major pursuit includes novel automated lesion segmentation methods for approved clinical use without necessary manual intervention. Recently, the first automated segmentation method using 67 segmentation masks from EpiBioS4Rx achieved 61% precision (Yildiz et al., 2022). Incremental improvements in automated methods can provide a starting point for students, which can greatly decrease the length of time needed for students to complete the initial segmentation. In this context, students would not need to start with a blank lesion mask and can use the generated masks as a guide for ROI identification.

Through the study of the described double review process, inter-rater reliability between the first and second reviewers was established (Bennett et al., 2022a,b). Moreover, the lesion core was identified as a more complex label to segment as DSC values for the initial segmentation to second review were significantly different between lesion core (62.54%) and total lesion volume (73.02%), which should be taken into consideration during manual segmentation and in the development of automated methods (Bennett et al., 2022a,b). Finally, it was suggested that our segmentation methods are robust between seizure and non-seizure groups as DSC values were not found to be significantly different between the two groups using a Mann–Whitney *U*-test (Bennett et al., 2022a,b).

### 5.4. Future works

With 127 segmentations now complete we intend to utilize the increased training set to maximize accuracy of machine learning models for segmentation tasks in TBI cohorts. Importantly, the development of a precise automated segmentation method using our gold-standard ground-truths will facilitate multiple avenues of PTE analysis, including unsupervised seizure classification and biomarker identification. However, supervised classification of PTE by applying machine learning models to the manually annotated MRI scans is also of clinical relevance. EpiBioS4Rx is an ongoing study, and we will acquire additional segmentation masks using the outlined methods in the future. Moreover, subjects enrolled in EpiBioS4Rx are in the process of PTE adjudication to confirm diagnosis, which will allow for robust lesion phenotyping for PTE biomarker identification.

## 6. Conclusion

PTE is a highly heterogenous disorder, and the underlying mechanisms are difficult to study. Lesion segmentations are a useful tool to characterize injury burden and identify potential imaging biomarkers of PTE. Automated segmentation methods have been shown to perform well in healthy subjects and in subjects with certain neurologic disorders. However, TBI-induced lesions are especially complex to segment without manual intervention. The protocol outlined in this work is necessary to create a robust dataset of hemorrhagic lesion segmentations in moderate-severe TBI patients. From this large dataset, we can better study PTE biomarkers, analyze inter- and intra- rater variability, and develop reliable automated segmentation techniques for clinical use.

## Data availability statement

The datasets presented in this article are not readily available because, the data described in this study is subject to the following licenses/restrictions: access to data must be requested and approved by the EpiBioS4Rx Steering Committee. Requests to access the datasets should be directed to epibiossteeringcommittee@loni.usc.edu.

## Ethics statement

The studies involving human participants were reviewed and approved by UCLA Institutional Review Board (IRB# 16-001 576) and the local review boards at each EpiBioS4Rx Study Group Institution. Written informed consent to participate in this study was provided by the participants' legal guardian/next of kin.

## Author contributions

AB composed the manuscript. PC, SG, AP, AK, and AB performed initial manual segmentations. AB, GB, RC, and JL performed initial reviews on the lesion segmentation masks. MM completed the final review of lesion segmentations. AB and CA conducted dice similarity coefficient analysis. All authors provided constant feedback and reviewed the manuscript. All authors contributed to the article and approved the submitted version.
