# Supplementary material for: Manual lesion segmentations for traumatic brain injury characterization
Source: Front Neuroimaging. 2023 Mar 16;2:1068591. doi: 10.3389/fnimg.2023.1068591 (PMC10406209; doi:10.3389/fnimg.2023.1068591)
Supplement: Supplementary file 1 [file Data_Sheet_1.docx]

EpiBioS4Rx: Tutorial for Manual Lesion Segmentation

**ITK-SNAP**

ITK-SNAP is a program used for manual segmentations, which will be used to segment lesions. It can be downloaded at this link:

<http://www.itksnap.org/pmwiki/pmwiki.php?n=Downloads.SNAP3>

**MRI Files**

In some cases, patients have both T2-FLAIR and T1-MPRAGE images available. Segmentations are performed on the T2-FLAIR scan. However, the T1-MPRAGE can be added as an additional image to aid in visualization of lesions when the contrast is poor, or the image has a lot of noise.

**Workspace**

Once downloaded, you can open the program and simply drag your file onto the screen to add the MRI image. Figure 1 shows what the window will look like once a T2-FLAIR image is opened in ITK-SNAP. There is an axial, sagittal, and coronal view. The A, S, and C in the top right corner of each individual window will remind you which image is which orientation. The bottom left window will show a 3D model of your segmentations if you select “update.”

Because each site operates a different scanner with slightly different parameters, each patient may have a different number of slices in one image. You can see the total number of slices in the bottom right corner of each view. You can also see which slice you are currently viewing. In the example below, the axial view is on slice 98 out of 160. To change which slice you are on, you can click the up and down arrows on the right-hand side or drag the bar up and down. Ensure you look through all slices to find all possible lesions in one scan.

**
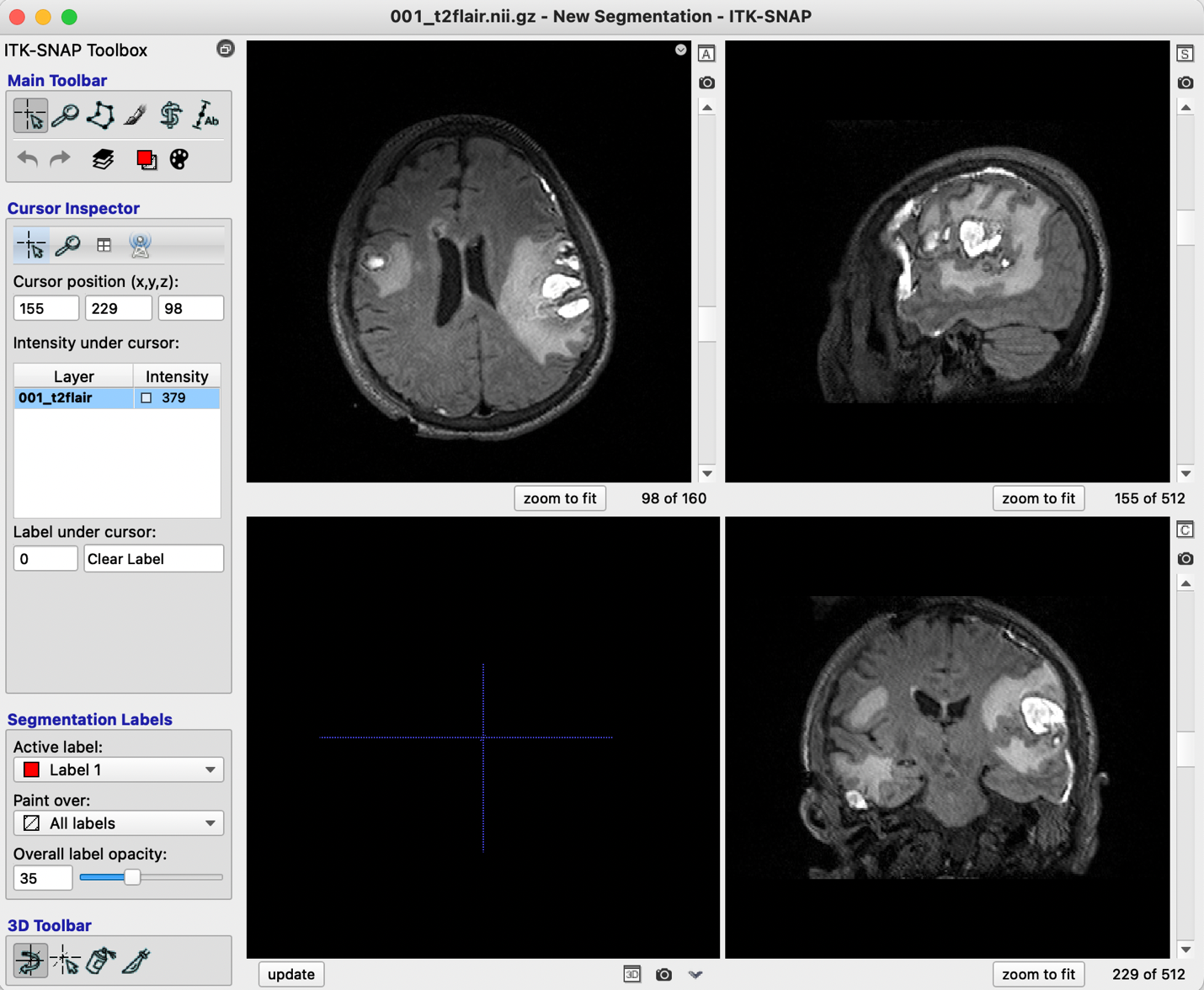
Figure 1:** New image opened in ITK-Snap prior to lesion segmentation

In the Main toolbar (Fig. 2) are different tools. For lesion segmentation, you will be mostly using the cursor icon (Crosshair mode) and the paintbrush icon (Paintbrush mode).


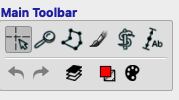


**Figure 2**: Various tools in the “main toolbar” of ITK-SNAP. The crosshair mode (circled in blue) and paintbrush mode (circled in red) will be the two main tools used for lesion segmentation.

**Crosshair Mode**

Crosshair mode is used to change your workspace view. In this mode you can zoom in and out, change the location, and move around the screen.

In this mode, if you click on any section of the brain in either view, it will automatically update your location in the brain in the other views.

To zoom in and out, hold *ctrl* while sliding your trackpad.

Once you zoom in or out, a yellow box will appear in the lower left-hand corner of your screen. If you hold your mouse around the box, it will slide the view of your scan around as well.

**Paintbrush Mode**

This is the mode to use when you are ready to begin segmenting. You can change your brush style and size in the left window (Fig 3). The circular brush style is often better for lesions. Before you begin segmenting, make sure your active label is correct (see Segmentation Labels section).


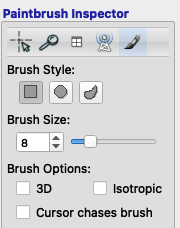


**Figure 3:** Paintbrush Inspector toolbox in which you can change your brush style and size.

*To segment*, simply left click on your mouse. You can hold and drag to do a large section at a time.

*To erase* any part of your segmentation, hold *ctrl* while holding your mouse over the section you want to erase.

When completing a segmentation, ensure you do so completely on one of the views (Axial, sagittal, or coronal). It does not matter which you choose but be sure to remain consistent. This will give a smoother and more accurate lesion segmentation once you are done.

After you completely segment the lesion and edema on one slice (ensure the lesion and edema are on different labels), you must go to the next slice and repeat this process until each slice with the lesion present is complete. This will give you the complete volume of the lesion.

**Differentiating Lesion vs. Edema**

Edema is the swelling due to excess fluid and often surrounds the core lesion. These two lesion types show up differently on an MRI. You will segment them on separate labels if they are differentiable.

On the scan, the lesion will usually appear white (or a lighter gray), while the edema appears light or dark gray. Almost always, edema looks darker. If it is hard to visualize, you can change the contrast of the image by going to *Tools -> Image Contrast -> Contrast Adjustment*. If you are still unable to visualize the lesion, or are having any difficulty locating lesions, reach out to other members in the lab who are familiar with lesion segmentation for clarity.

Here is an example of lesion vs. edema segmentation on one MRI slice, with lesion in red and edema in green (Fig. 4).


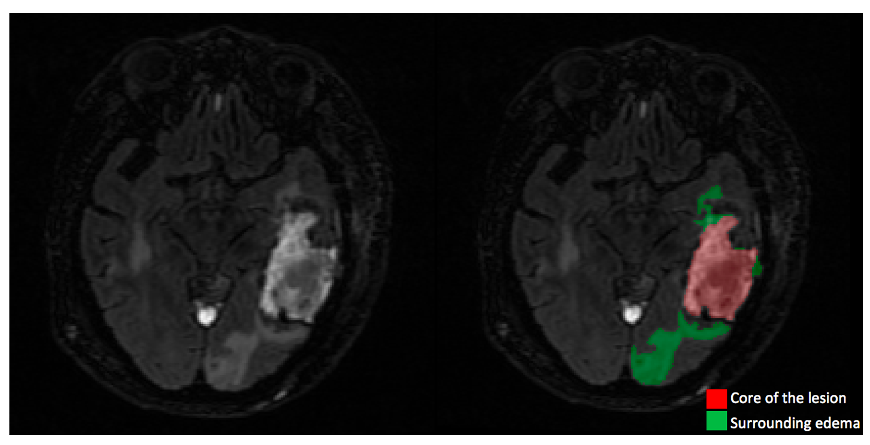


**Figure 4:** One MRI slice before (left) and after (right) lesion segmentation. The core lesion is segmented in red and surrounding edema is segmented in green.

**Segmentation Labels**

To change your active label, use the box in the lower left corner of your workspace. Lesion and edema will be on separate labels. For example, Label 1 (red) would correspond to lesions and Label 2 (green) would correspond to edema. It may also be useful to use the Clear Label if you are not able to erase your segmentation using the *ctrl* key in Paintbrush mode. Going over a portion of your segmentation while using the Clear Label is an alternative way to erase any mistakes.

To change between labels, click the dropdown under Active label (Fig 5).

To change the color or names of each label, you can go to Segmentation -> Label Editor in the top bar.


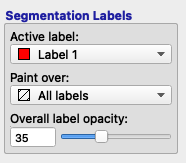


**Figure 5:** Tools to adjust segmentation labels. The “Active label” dropdown bar will allow you to switch between labels.

**Recording Lesion Volumes:**

After a lesion segmentation is complete, it is important to record the volume of each lesion and its corresponding edema for future analysis. To do so, go to Segmentation -> Volumes and Statistics from the top bar. The “Volumes and Statistics” window will appear (Fig. 6).

**
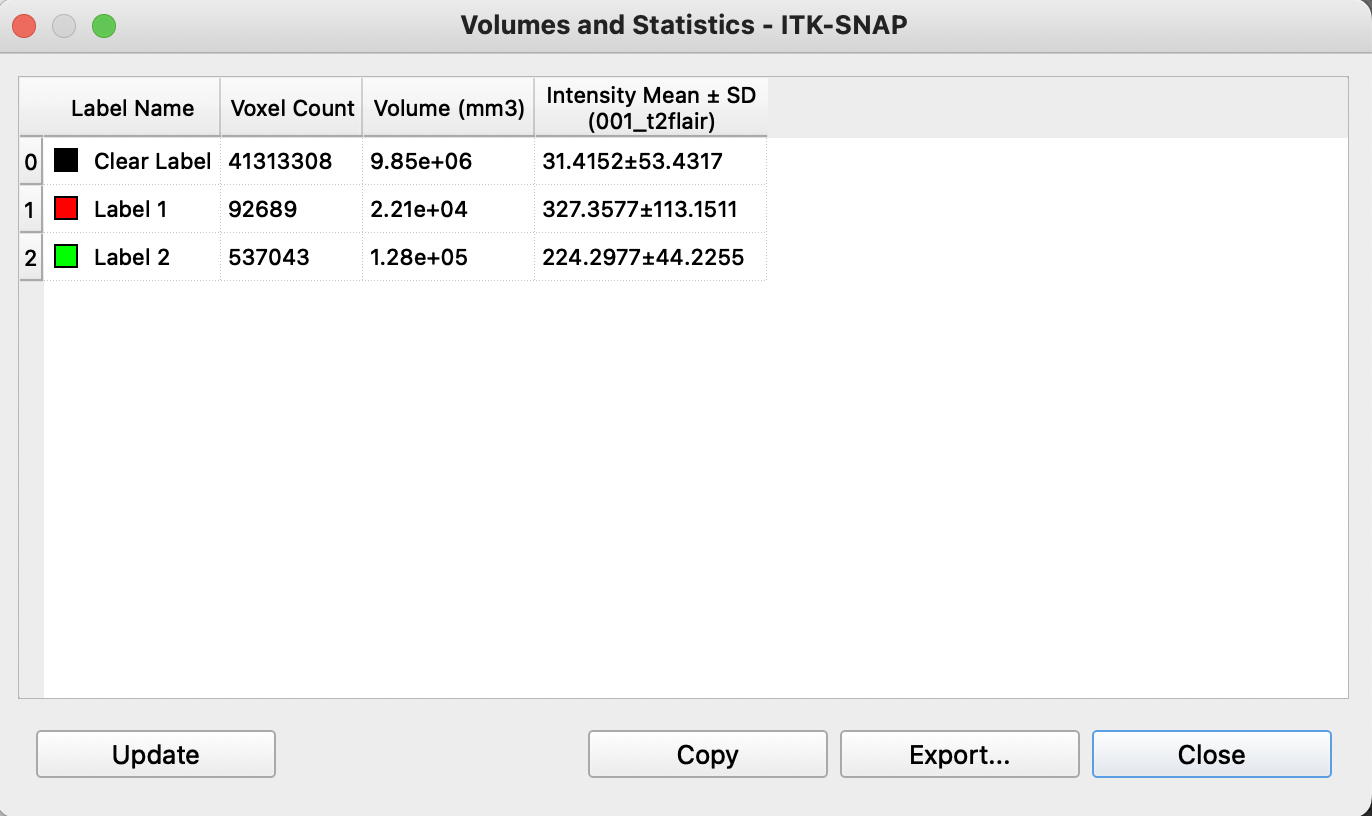
**

**Figure 6:** The “Volume and Statistics” window which will provide volumes for all labels**.**

Volumes will be separated by label. In an excel sheet, record the values from the Volume (mm3) column. In this example, the lesion volume (Label 1) would be 22100 and the edema volume (Label 2) would be 128000.

Figure 7 displays an example of complete volume entries for two patients. You should record individual lesion/edema volumes as well as the total volume across each patient. For easier analysis, also include the general area the lesion is located in (Left/Right hemisphere + Lobe).

*Reminder: L/R in ITK-SNAP is in radiologic view, so it is the opposite of left vs. right on the screen.

**
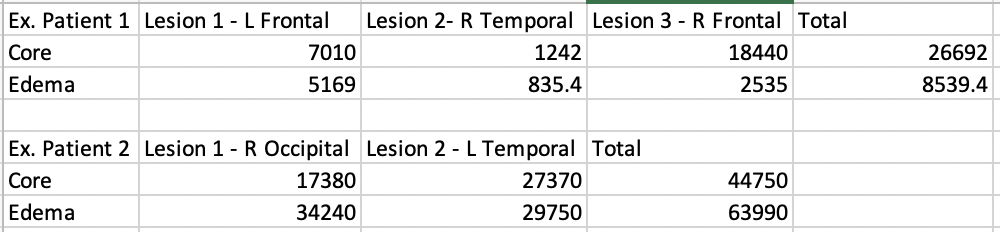
**

**Figure 7:** Two complete patient entries in an excel sheet. Volumes for both lesion and edema for individual lesions as well as the total volumes across each patient are recorded.

**Saving Segmentations:**

After completing one lesion segmentation, be sure to save your work. To save, you can either use *ctrl + s* on the keyboard or Segmentation -> Save Segmentation Image from the top bar. When saving, use the same formatting as the original file name and add the lesion number at the end.

Ex. The segmentation file for lesion 1 on example patient 001 on 001_t2flair.nii.gz would be 001_seg_lesion_1.nii.gz

**Segmenting Multiple Lesions:**

Some patients may only have one lesion. However, if one patient has multiple lesions, you must segment them all separately and save them as separate files. To begin a new lesion, redrag the MRI image into ITK Snap and select Load as Main Image. This will give you a clean image to start segmenting a new lesion. Each lesion will also be recorded as a separate volume on the excel sheet.

**Common Commands on Mac:**

*S* : Set label opacity to 0 or return it back to normal level

*A* : Decrease label opacity by 5

*D* : Increase label opacity by 5

*Ctrl + hold mouse* : When in Paintbrush mode, this is how to erase. It will only erase the color of your active label.

*Ctrl + hold + drag :* When in Crosshair mode, this will zoom in and out

*Ctrl + z* : Undo
